# Supplementary figures and images for: The Occurrence and Meta-Analysis of Investigations on Sarcocystis Infection among Ruminants (Ruminantia) in Mainland China
Source: Animals (Basel). 2022 Dec 30;13(1):149. doi: 10.3390/ani13010149 (PMC9817671; doi:10.3390/ani13010149)

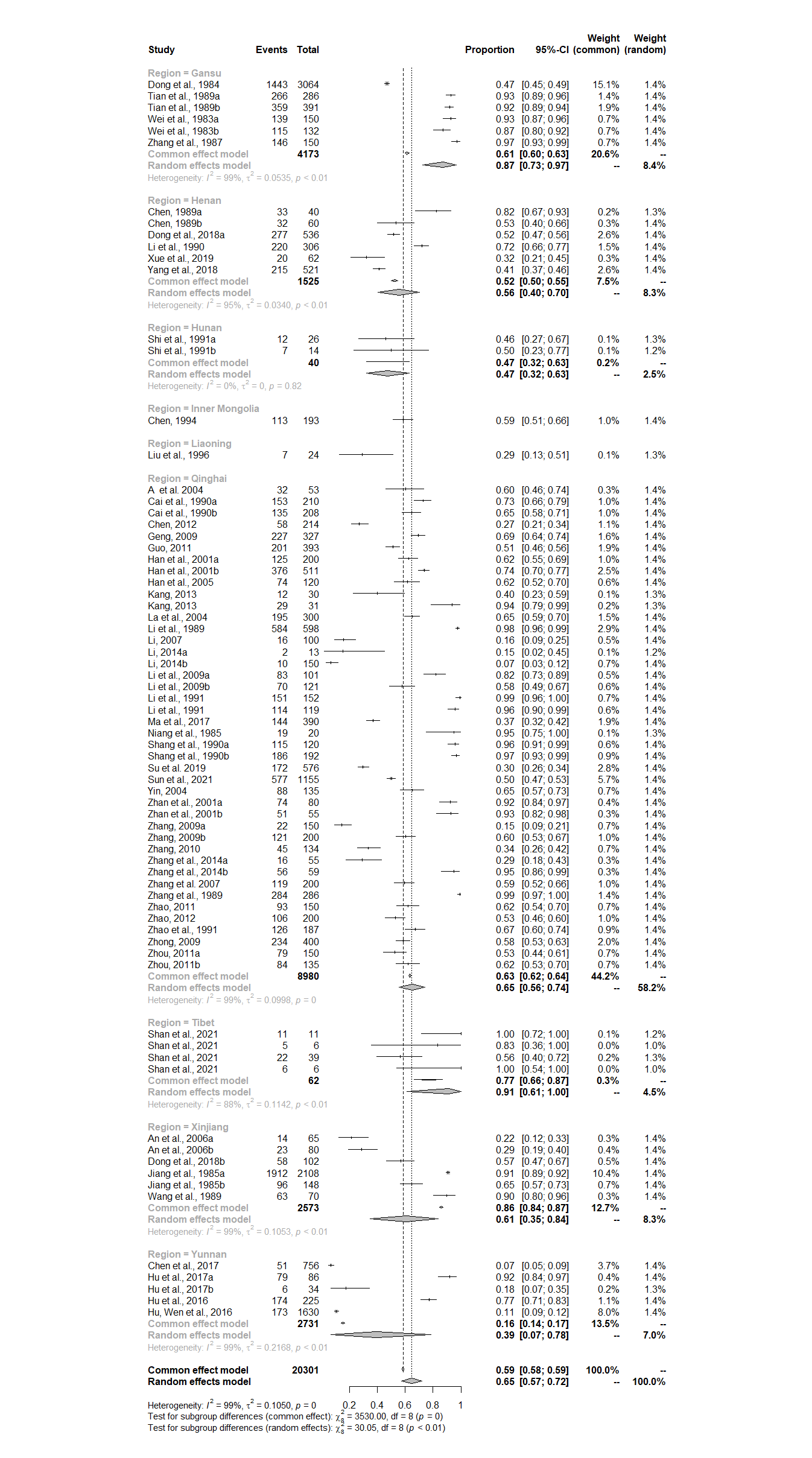

Supplement: Supplementary file 1 [file animals-13-00149-s001.zip › Figure S1.tiff]

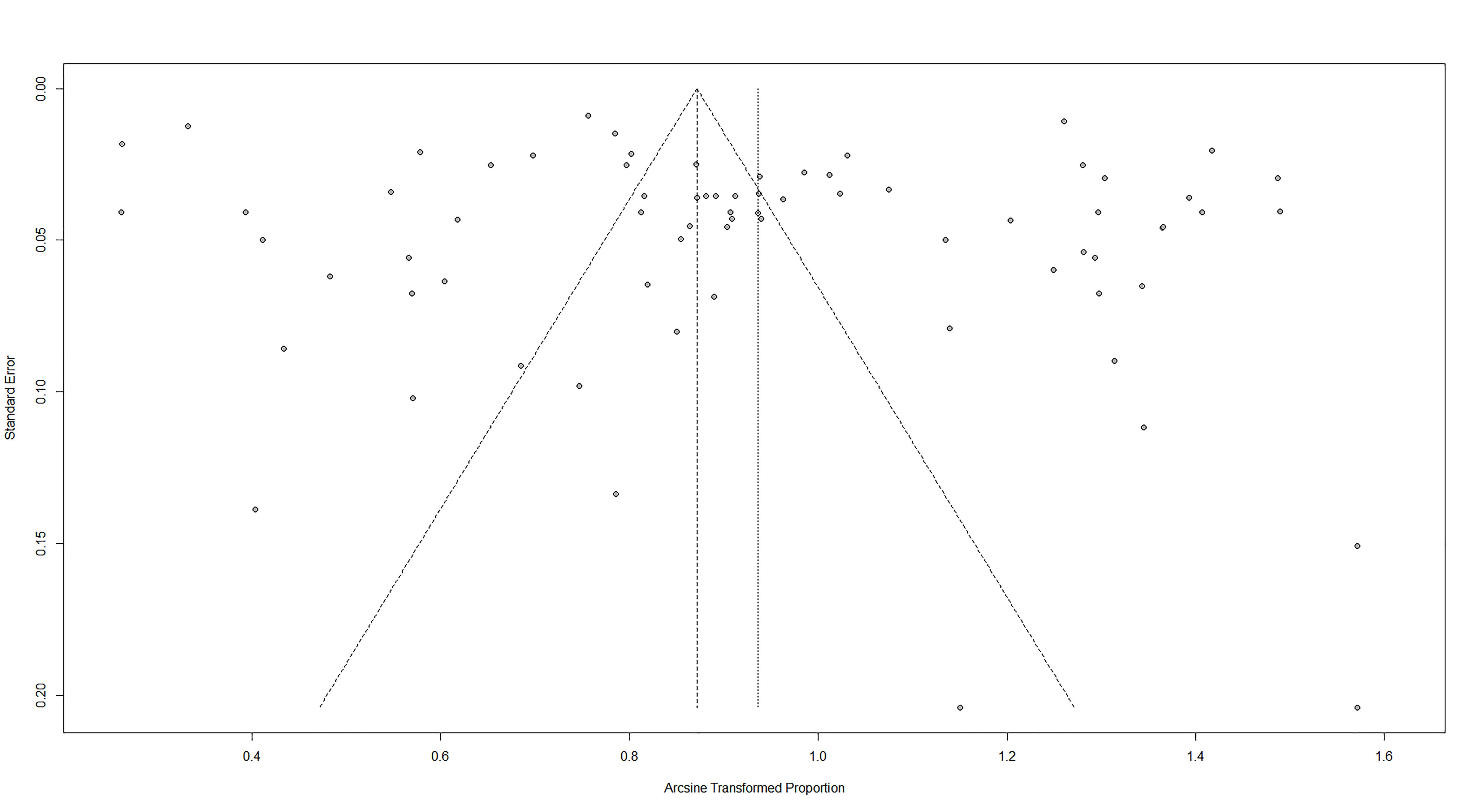

Supplement: Supplementary file 1 [file animals-13-00149-s001.zip › Figure S2.tif]

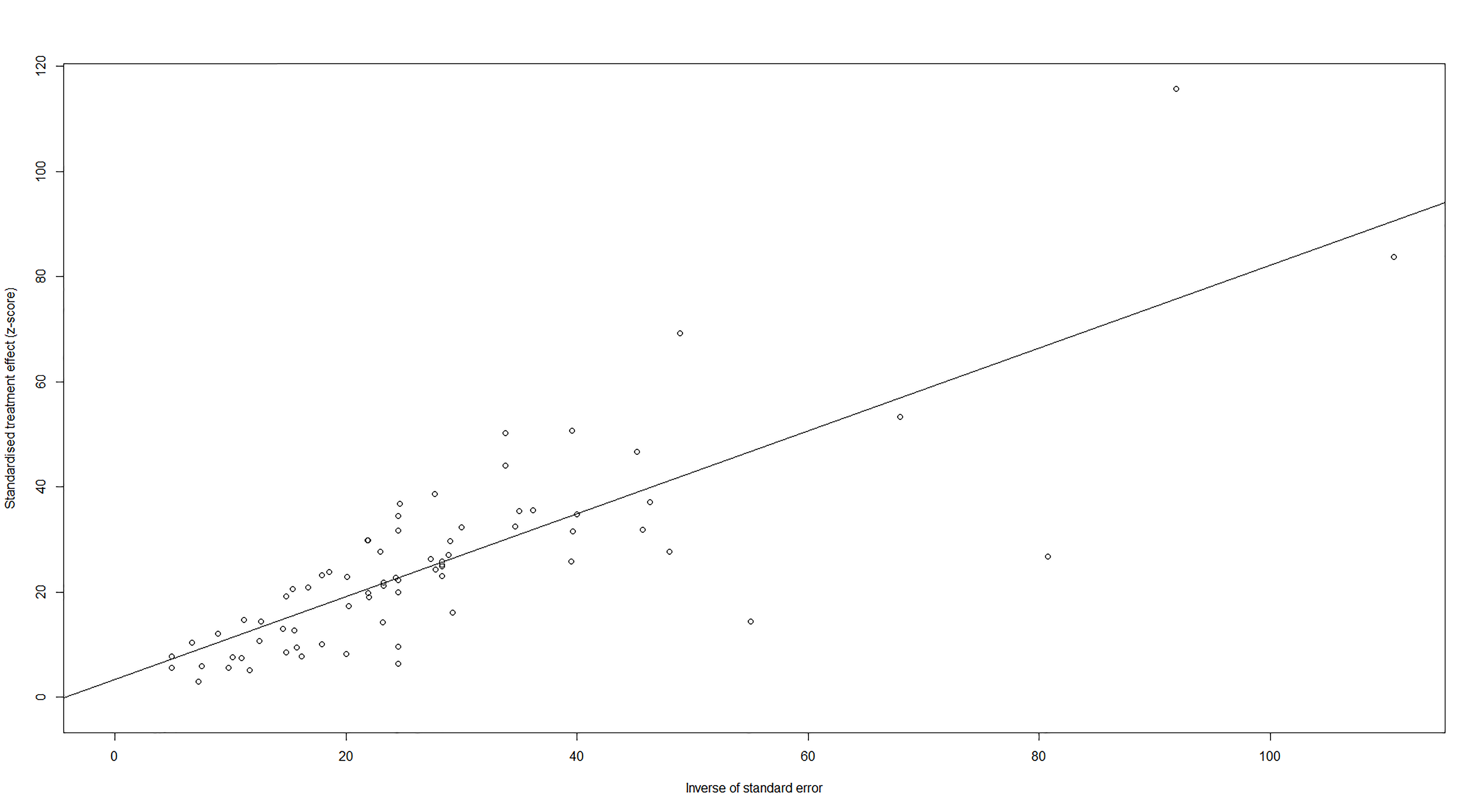

Supplement: Supplementary file 1 [file animals-13-00149-s001.zip › Figure S3.tif]

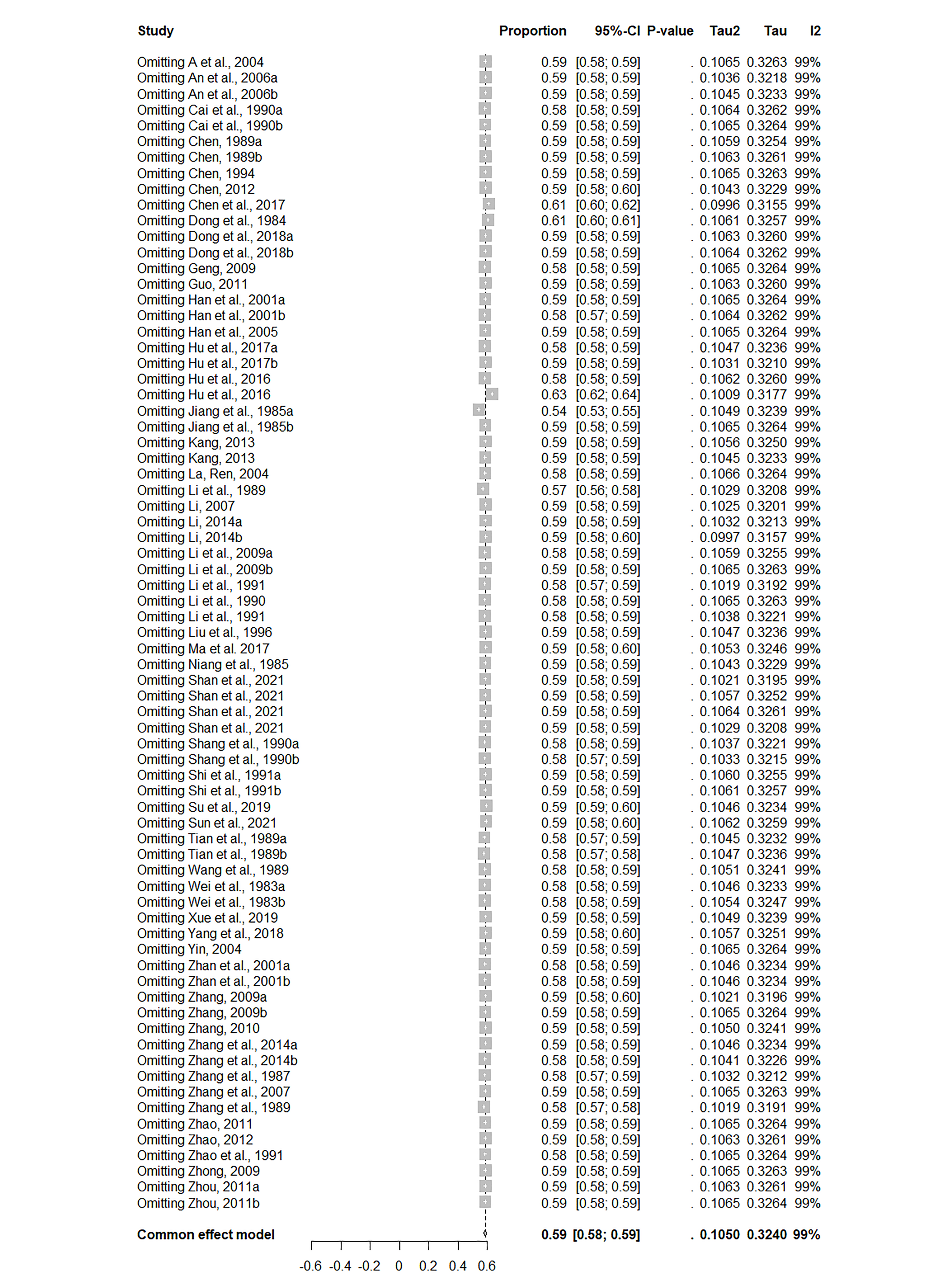

Supplement: Supplementary file 1 [file animals-13-00149-s001.zip › Figure S4.tif]

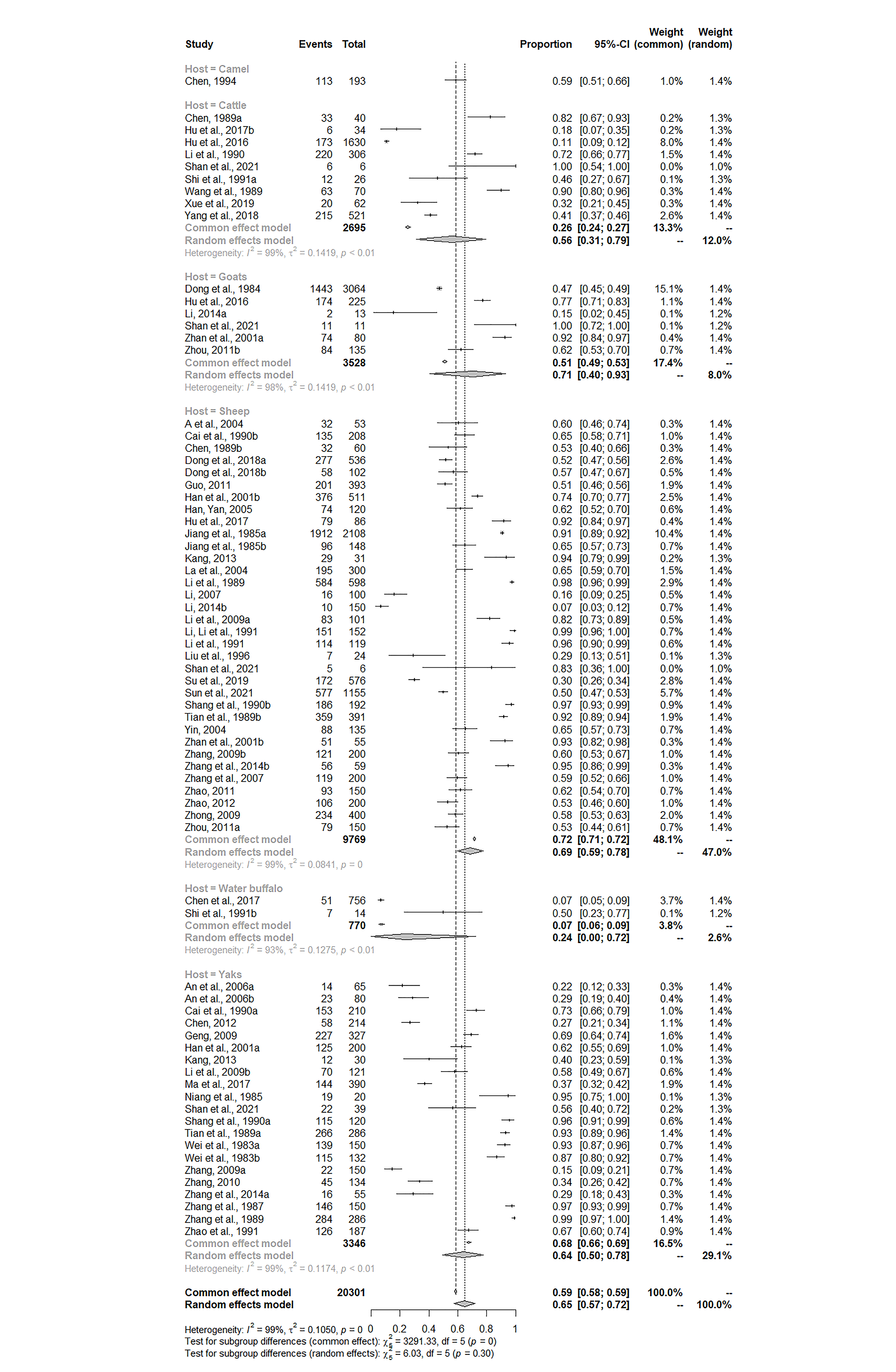

Supplement: Supplementary file 1 [file animals-13-00149-s001.zip › Figure S5.tif]

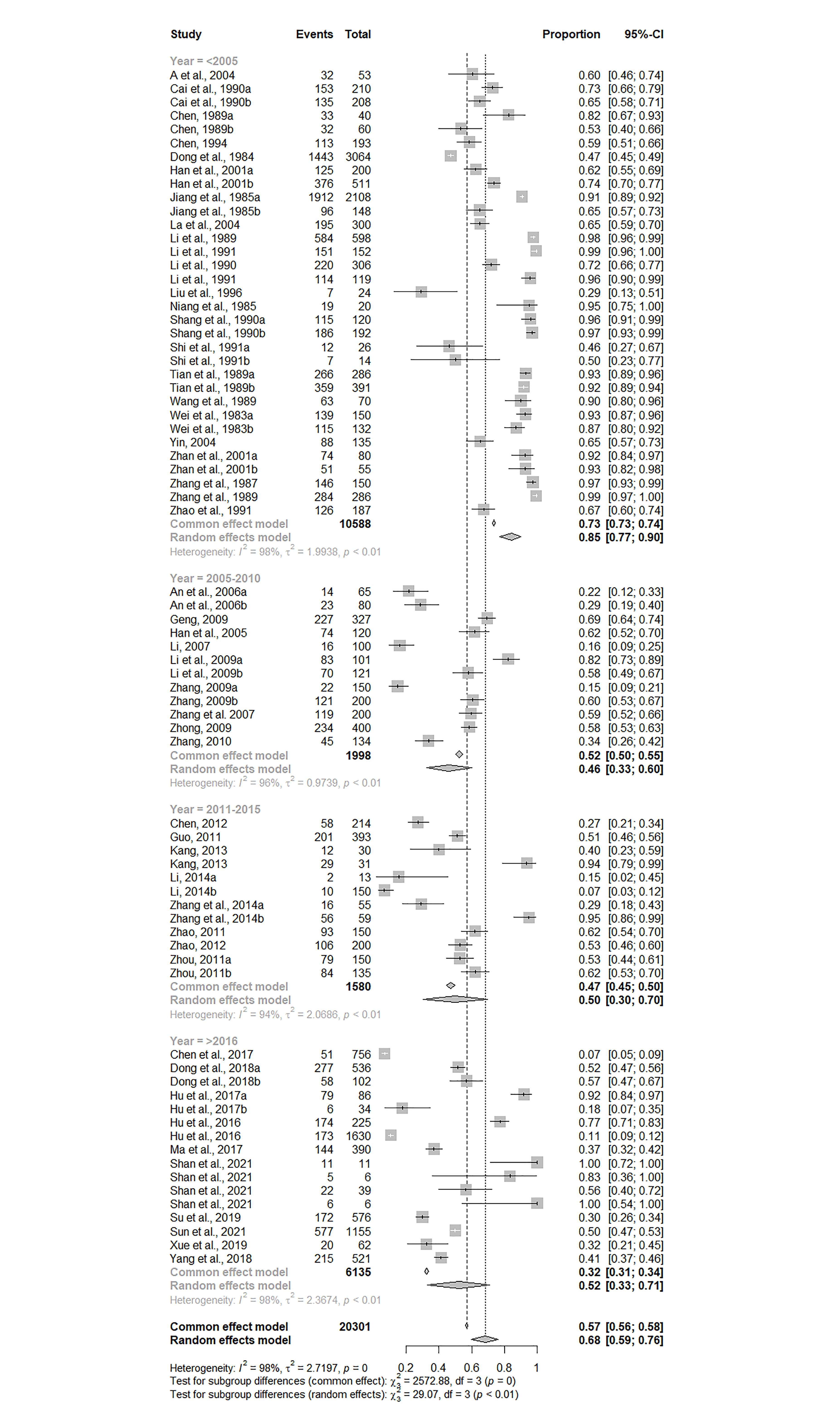

Supplement: Supplementary file 1 [file animals-13-00149-s001.zip › Figure S6.tif]
